# Complementary Study Based on DFT of Optical and Electronic Properties of New Copolymer PVK-F8T2

**DOI:** 10.3390/polym13111805

**Published:** 2021-05-30

**Authors:** Yasmine Ben Salah, Abeer S. Altowyan, Mohamed Mbarek, Kamel Alimi

**Affiliations:** 1 College of Sciences of Monastir, Monastir University, Monastir 5000, Tunisia; yaminebensalah94@gmail.com (Y.B.S.); kamel.alimi@fsm.rnu.tn (K.A.); 2 Department of Physics, College of Science, Princess Nourah bint Abdulrahman University, Riyadh 11671, Saudi Arabia

**Keywords:** energy transfer, optical transient, optoelectronic, OLED

## Abstract

This article is mainly a complementary study of a novel part of π-conjugated copolymers based on the poly (N-vinylcarbazole) (PVK) and poly (9,9-dioctylfluorene-co-bithiophene) (F8T2) unit based on the Density Functional Theory (DFT) and Time-Dependent Density Functional Theory (TD-DFT). This study is carried out to explore the structural and optoelectronic characteristics of a new organic material named PVK-F8T2. First, the structural, optical (absorption, photoluminescence, optical transition), electronic (molecular orbital (MO), energy-level diagram) and vibratory parameters of infrared (IR) were computed and compared with experimental studies. In addition, we calculated the level energy of the excited states and their corresponding transitions. Obviously, electronic parameters such as highest occupied and lowest unoccupied molecular orbital (HOMO and LUMO), ionization potential (IP), electronic affinity (EA) and the energy band gap (Eg) were computed in order to elucidate the intramolecular charge transport and to establish the energetic diagrams of the PVK-F8T2 copolymer for different states. The results obtained looked with precision at future optoelectronic applications. From these results, we have shown that the PVK-F8T2 has significant optoelectronic properties and seems usable as an active layer in organic light-emitting diodes (OLEDs).

## 1. Introduction

Like many other important discoveries in the science field, the progress of the organic electronic sector based on π-conjugated polymers is taking an increasingly interesting place in our daily life. As the operational and functional devices have already been launched, the key issue is to quickly optimize and improve their performances. In order to achieve this, it becomes very useful to engineer new materials whose optoelectronic characteristics are original and competitive.

Indeed, polymers have many applications, such as OLED [1,2,3] and organic photovoltaic (OPV) [4,5,6,7], because of their ease of shaping as well as their low production cost. For this reason, the current scientific and industrial challenge is to design new ideally soluble [8] conjugated polymers with the aim of obtaining a new material having modular optoelectronic properties endowed with the desired performance, thus improving their solubility [9,10] in order to act as active layers in optoelectronic devices [11,12,13]. Research in these directions has been fruitful previously in polymers such as the PVK [14,15,16,17,18,19,20] and the F8T2 [21,22,23], which have become successful in recent years thanks to their characteristic properties. To this end, considerable efforts have, therefore, been allocated to design and elaborate on this new copolymer PVK-F8T2. The resulting copolymer with desirable properties has been tested and investigated experimentally [24,25,26]. Presently, computed investigations have become efficient methods to check out the optoelectronic properties of the active layer operating in organic devices. DFT calculations are one of the solutions performed to conceive the properties of the active layers in organic devices supporting efforts to advance the design and efficiency of devices. Additionally, they are employed to govern a comparative analysis of their experimental and theoretical results based on the same material. It makes it possible to establish a strong coupling with experimental data, leading to structure–property correlations.

In contempt of the several methods for the experimental characterization of the new PVK-F8T2 copolymer, conformational and optoelectronic information remains difficult to study. To this aim, a complementary study based on the DFT was executed in order to more understand the structure–property relation and to optimize their optoelectronic applications [27]. In this context, to verify the possibility of applying a new copolymer as an active layer in electronic devices, a thorough appreciation and restrain of the relationships between property and structure are necessary. For this goal, DFT calculations were achieved to further understand their main characteristics of PVK-F8T2 and to upgrade the design and optimize the performance-based devices.

In order to achieve this, we studied the optimized model structure of PVK-F8T2 from ground states to excited states, including a conformational study, spin density and possible scenario of the copolymer growth, charge distribution, optical transients, and finally, electronic structure and energy diagram of the electronic device founded by PVK-F8T2. This complementary study aims to describe deeply the molecular structure–property relationship; we used all the obtained conclusions to search for a finer explanation of the optoelectronic properties of the copolymer. The present works aims to give us some optoelectronic characteristics of the PVK-F8T2 based on DFT calculations, such as electronic structure and optical transitions, and to test the copolymer in an electronic device and optimize its application.

Is this a donor/acceptor (D/A) system? Is the coupling between homopolymers PVK and F8T2? What is the electronic structure? Which blocks are responsible for optical transitions? Is this PVK-F8T2 suitable for optoelectronic applications?

In light of these results, we will propose exploitable model structures for optoelectronic applications with optimal properties.

This study allowed us to describe the conformity and the optoelectronic properties of PVK-F8T2 where calculations were carried out at the station installed in our laboratory via the Gaussian 09 [28]. This new theoretical study is based on the DFT and TD-DFT calculations implanted in Gaussian via the basis B3LYP 6-31 G (d.p) [29,30,31,32,33,34]. This basis takes into account both polarization and diffuse functions and includes the effects of electronic correlation for a lower cost in computing time. The computed results are then confronted with experimental data. The representation of molecular orbitals was performed using the Gauss View graphical interface (5.0).

Some electronic parameters are deduced based on some mathematical formulas:

Eg_elec_ = E_HOMO_ − E_LUMO_ (1)

(2) $${\mathrm{Eg}}_{\mathrm{opt}}=\frac{1240}{\lambda }$$

Based on the Koopmans theorem IP (ionization potential) = −E_HOMO_; EA (electron affinity) = −E_LUMO_

## 2. Experimental and Theoretical Results

It is important to recall that the PVK-F8T2 was synthesized via an oxidative pathway using FeCl_3_. F8T2 (M_n_ 20,000) and PVK (M_n_ 25,000–50,000) (purchased from Sigma Aldrich, St. Louis, MO, USA) were dispersed in chloroform and added with FeCl_3._ The prepared mixture was kept under reflux heating at 55 °C for 3 days. After that, the PVK-F8T2 copolymer was rinsed and filtered by methanol. A better description of PVK-F8T2 preparation was exposed in the previous paper [24].

A scanning electron microscope SEM (JEOL, JSM-7600F operating at 5 kV, (IMN, Nantes, French) was utilized to explore the structuring of the PVK-F8T2. The infrared absorption curve was registered with a Brüker Vector 22 Fourier transform spectrophotometer (FSM, Monastir, Tunisia). UV–vis absorption was recorded with a Perkin-Elmer (Lambda 1050) spectrophotometer (IMN, Nantes, French). Emission was examined using a Horiba Jobin-Yvon Fluorolog 3 equipped with a CCD camera (IMN, Nantes, French).

Relying on the experimental study [24,25,26] realized during the preceding works, we carried out a primordial study on the N-vinylcarbazole dimer via spin density and dihedral angles (scan). We can subsequently define the scan as the computation of the relative energy as a function of the angle of twist between two planes of two units of attached polymers.

**Figure 1:**
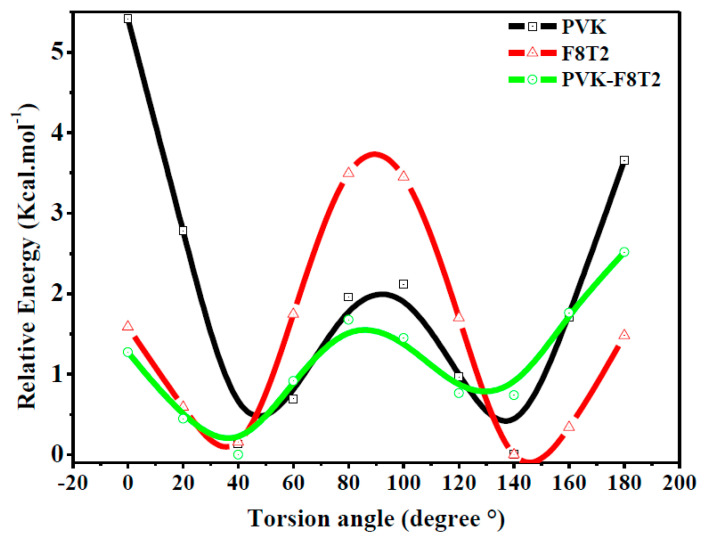
Energy curve of PVK, F8T2 and PVK-F8T2.

In fact, we varied the angle of twist between the two-carbazole units from 0 to 180° with a step of 20° to ensure the stability of the bicarbazole unit. The obtained result from Figure 1 shows that the most stable state corresponds to the angle θ = 140° (40°) of the most stable ground state. As a matter of fact, the angle of torsion established a compromise between the effect of conjugation, which promotes a planar structure, and the effect of steric repulsion between hydrogen atoms, which promotes a non-planar structure, as has been discussed in previous work [35,36].

In order to conceive the grafting sites of F8T2 in the PVK, a spin density calculation of bicarbazole formed following the oxidation reaction was carried out [37].

**Figure 2:**
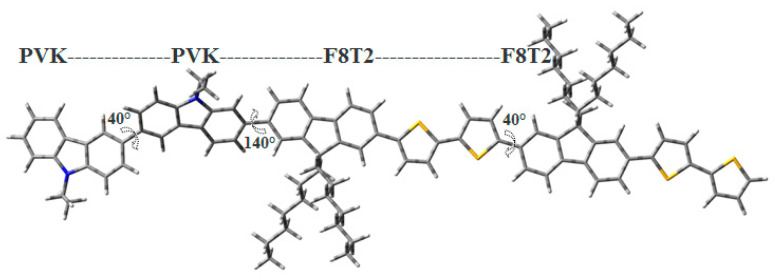
Chemical structure proposed for the PVK-F8T2 copolymer.

We recall that the spin density calculation was performed after optimization in the oxidized state. It is important to take into account that the experimental infrared curve of the PVK-F8T2 has shown a formation of bicarbazole units following the oxidation reaction using FeCl_3_, which is known as cross-linking phenomenon [38,39]. Thus, the suggested model structure of PVK-F8T2 is comprised of two carbazoles (PVK)_2_ units with a dihedral angle around 40° linked to a chain or chains of F8T2 with a dihedral angle around 140°, as shown in Figure 2.

**Figure 3:**
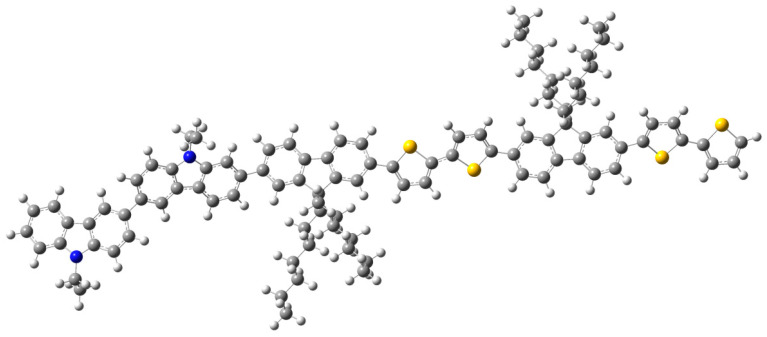
Optimized model structure of the PVK-F8T2.

With the aim of verifying the authenticity of the model found, several models are proposed and tested while keeping, in the model structure, the two carbazole units and varying the number of units of F8T2, ranging from one to four units. Therefore, the performed series of calculations show that the model structure shown in Figure 3 is composed of a carbazole block (two units) linked to the F8T2 block (two units). To find the structure–properties correlation of the PVK-F8T2, we combined the experimental and calculations results.

The model structure obtained was first optimized in the fundamental state via exerting the B3LYP/6-31G (d.p) [40].

**Figure 4:**
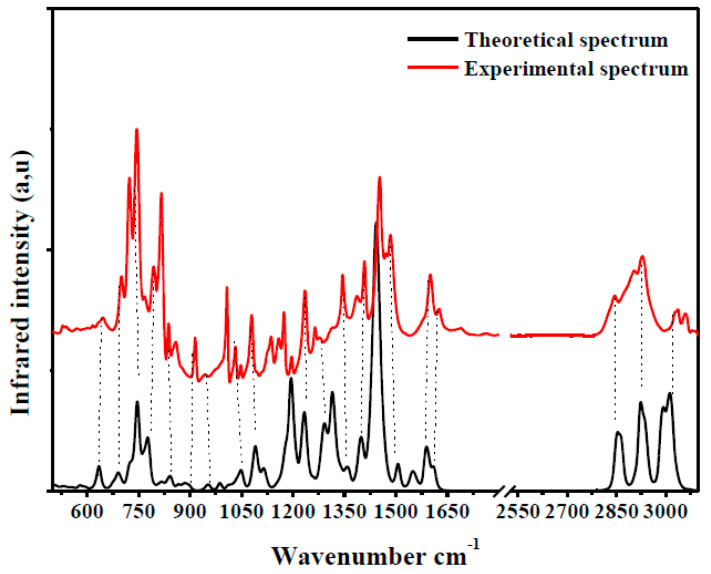
Experimental and simulated infrared spectra of PVK-F8T2.

To validate the model structure, we simulated the infrared spectrum of the copolymer. The experimental and theoretical infrared spectra are shown in Figure 4.

The two IR curves have the same shape in terms of bands, positions and intensities. In parallel, it is noted that the experimental characteristic bands of the copolymer (787, 1355, 1592 and 3396 cm^−1^) appear in the simulated spectrum with a slight shift of a few bands that do not exceed 20 cm^−1^. This shift is due to the optimized structure in the gaseous state, where the intermolecular interactions present in the condensed state are strong, while they are absent in the gaseous state. From this agreement of the two spectra, we can conclude that the deduced model structure reproduces the vibrational modes of the synthesized material. This validates our preference for the model structure.

**Figure 5:**
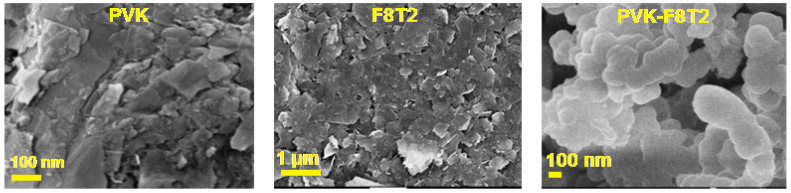
SEM Images of PVK, F8T2 and PVK-F8T2.

The morphology of the two base homopolymers PVK and F8T2 and the formed copolymer PVK-F8T2 has been investigated by SEM. The SEM micrographs are reported in Figure 5.

The dramatic change in morphology allows PVK and F8T2 to become the PVK-F8T2 copolymer. A condensed globular morphology was observed in PVK-F8T2, referred to as the stacked-layered surface of PVK and F8T2. This new morphology is attributed to the linking and the functionalization of PVK and F8T2 and the forming of the PVK-F8T2 copolymer.

**Figure 6:**
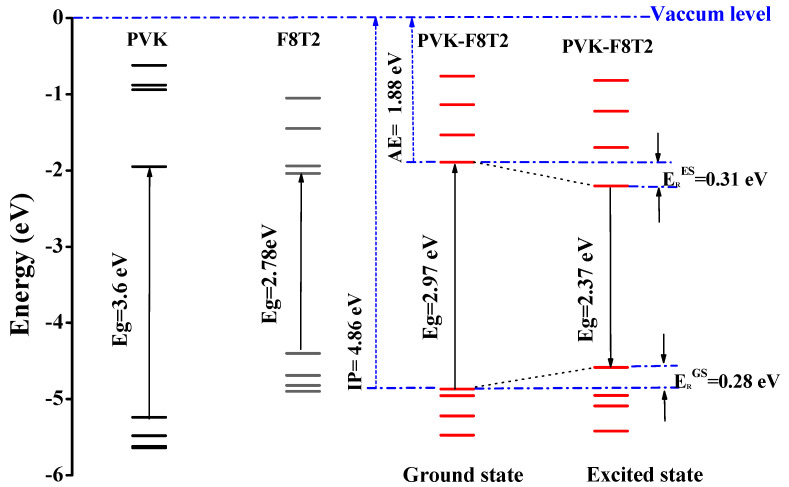
Electronic diagrams of PVK, F8T2 and of PVK-F8T2.

The electronic structure of PVK-F8T2 in the ground and excited state are shown in Figure 6. The HOMO and LUMO energy levels are known to be immediately connected to the electronic band gap Eg ionization potential (IP) and electronic affinity (EA), respectively [41,42,43].

The E_g_^Elec^ of the PVK-F8T2 (2.37 eV) shows a reduction in the fundamental and the excited state compared to the PVK (4.4 eV). This indicates that an intra-molecular charge transfer occurs between the PVK block and F8T2 with the electronic exchange between them [25].

To provide a qualitative description of photogenerated charge transport, we have examined the HOMO and LUMO in both ground and excited states. At the ground state, the HOMO and LUMO have the respective values −4.86 and −1.88 eV. However, in the excited state, these values are evaluated at −4.58 and −2.2 eV, respectively. Regarding the change of the LUMO, after excitation of the copolymer, it has been stabilized at approximately 0.27 eV, while the HOMO has undergone a slight destabilization of approximately 0.01 eV. The IP and EA are computed to judge the energy barrier for the injection of the two holes and of electrons in the PVK-F8T2. We can note that the energy needed to appoint a hole is 4.86 eV, as shown in Figure 6, while the removal of an electron necessitates energy of 1.88 eV. We can also notice that after excitation, the copolymer has lost part of its energy, which is also called relaxation energy. This energy is expressed by *E_R_* = $E_{R}^{GS}+E_{R}^{ES}$, which has the value of 0.59 eV. Hence, we suggest that a charge transfer occurs from bicarbazole units to F8T2 after excitation. We can imagine that the F8T2 blocs dome the electron density delocalization rather than PVK blocs.

**Figure 7:**
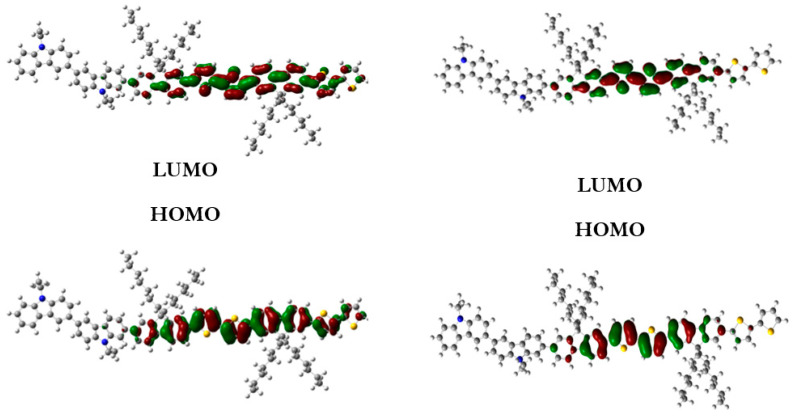
MO of the PVK-F8T2 in the ground and excited states.

To defend this reasoning, a plot of molecular orbital contour (MO) is presented in Figure 7.

Scanning of the molecular orbitals of this copolymer revealed that the electron density of the HOMO and LUMO orbitals in both ground and excited states are dominated by the block of F8T2. This can be assigned to the occurrence of charge/energy transfer from PVK to F8T2 blocks [42]. The contours of MO in the excited state signify the richness of electronic density in the F8T2 blocks, which have the major contribution of the emission due to the π system conjugated on them.

**Figure 8:**
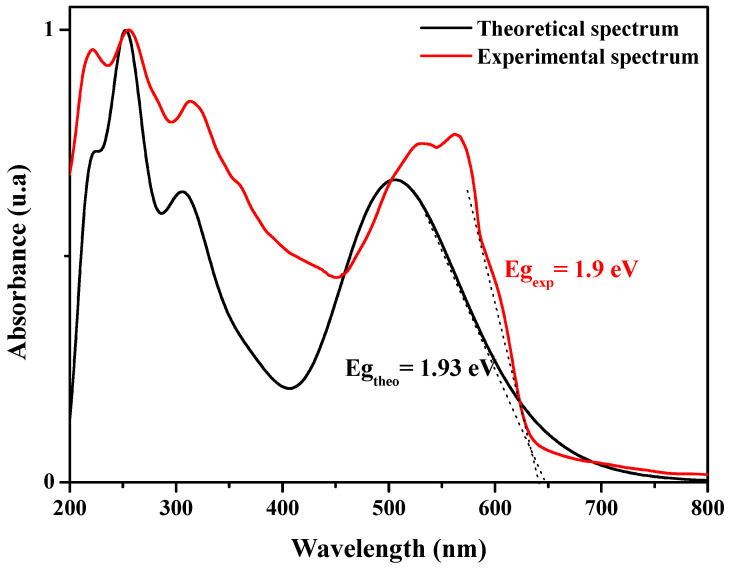
UV-vis absorption spectra of PVK-F8T2: experimental and theoretical.

It is important to examine the optical electronic transitions of absorption (Abs) and photoluminescence (PL) of the PVK-F8T2. In Figure 8, the analysis of the theoretical and experimental absorption spectra shows consistency between them.

A cool accord between the experimental and computed UV–vis absorption curves of the PVK-F8T2 is pronounced. As well as the position of the optical bands, we can notice that the simulated UV–vis absorption spectrum has five bands (222, 252, 306 nm, a shoulder at 358 and 507 nm). The two bands, 252 and 306 nm, are allocated to the n→π* transition. The peak located at 507 nm is attributed to the π→π* transitions [39]. It is important to mention that the theoretical spectrum appears to be shifted towards the red, in particular the last band. This is due to the absence of inter-chain interactions in the gaseous state and the isolation of the copolymer chain in the case of calculation. The three optical transitions and their attributions are shown in Table 1 for the neutral state.

As a general rule, the tolerance between the theoretical gap calculated for a chain isolated in the gaseous state and that evaluated experimentally in the solid state is evaluated around 0.3 to 0.4 eV [39]. Even so, intermolecular effects cannot be negligible in polymeric materials. By taking into account these impacts, the calculated results reproduce the experimental ones that confirm the resemblance of the theoretical spectrum with the experimental one, again validating the model structure, which still reproduces the properties of the copolymer.

**Figure 9:**
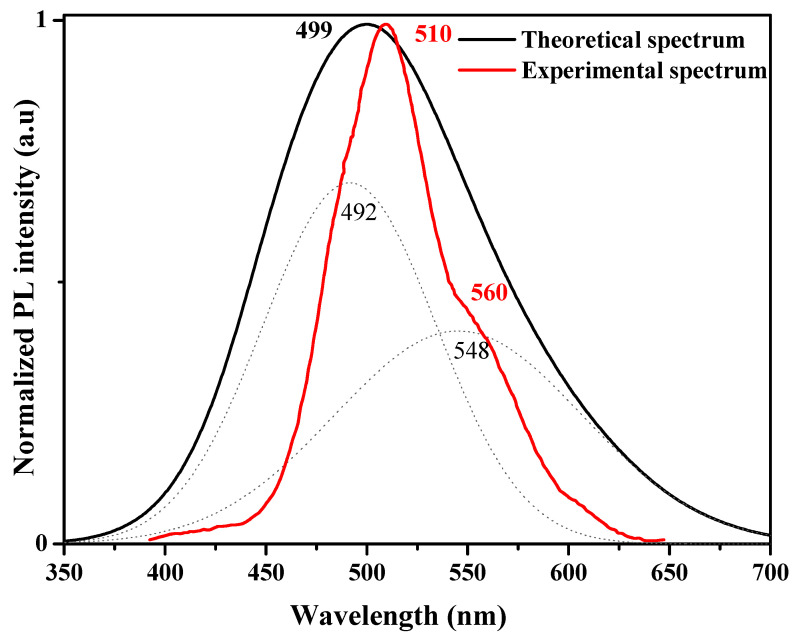
PL spectra of PVK-F8T2 copolymer.

The excited state of PVK-F8T2 and their corresponding optical transitions are computed by the TD-DFT method. Figure 9 displays the simulated PL of PVK-F8T2 compared to the experimental one.

From this curve, we can see that the calculated spectrum well reproduces the experimental one (in particular the λ_max_), which proves the selection of the structure-model proposed previously. The theoretical PL λ_max_ located at 499 nm was very close to the experimental one at 510 nm. This very weak difference is attributed to the negligible interchain interaction and the DFT calculations. Two peaks at 492 and 548 nm can fit the calculated curve, which are very close to experimental peaks found at 510 and 560 nm. From the value of the emission oscillator force at various excited states illustrated in Table 2, we can estimate the radiative lifetime (τ) of each transition using the Einstein transition probabilities [39].

(3) $$\tau ={\frac{c^{3}}{2f{\left(E_{fluo}\right)}^{2}}}^{}$$

where *c* is the speed of light, *E_fluo_* is the *E_max_*, and *f* is the oscillator force.

In Table 2, the calculations give a PL_max_ at 499 nm attributed to the S1→S0 (98%) transition with an oscillating force of 2.2, which indicates an intense fluorescence of the excited state. We can also see that the computed transitions bands of the PL are very close to the experimental ones.

In a sequence of valuing the energy barriers of the electron injection and the holes between the PVK-F8T2 and the metal electrodes, their work output and the HOMO and LUMO energy of the PVK-F8T2 are recorded in Table 3.

**Figure 10:**
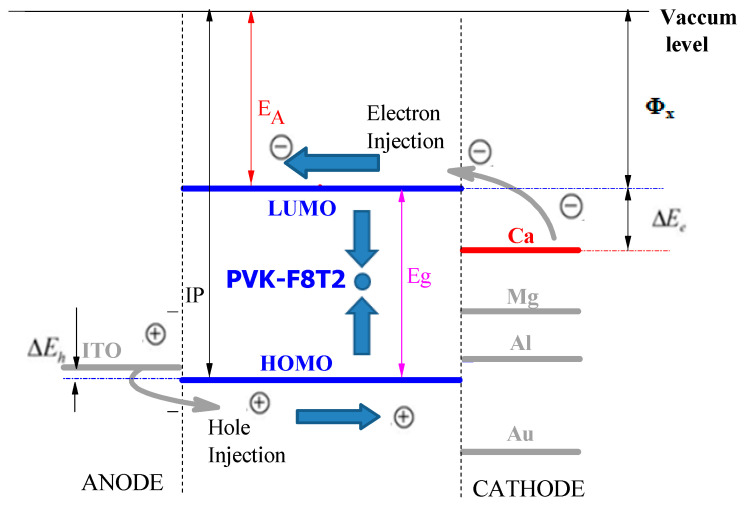
The active layer energy barriers with ITO/PEDOT:PSS/PVK-F8T2/Ca, Ag, Al, Au. (PEDOT:PSS: Poly(3,4-ethylenedioxythiophene)-poly(styrenesulfonate)).

We recall that the ITO anode output work is around 4.8 eV, the hole injection barrier is mentioned by ΔE_h_ = E_HOMO_ − 4.8 eV and the electron injection barrier is exhibited by ΔE_e_ = Φ_x_ − E_LUMO_, where Φ_x_ is the output work of the cathode. The difference (ΔE_e_ − ΔE_h_) will be helpful to identify a low output-work metal. Pursuant to this table, the optimization shows that the Ca and the Mg are the most desirable electrodes to diminish the electron injection barrier and holes [39] for the PVK-F8T2 (Figure 10).

## 3. Conclusions

To better understand the structure–property relationship, a combination of the experimental study with the theoretical one based on DFT calculations was prepared. The latter enables finding a model structure from which we have been able to determine the optical properties (UV–vis absorption, IR and PL), the electronic structure and the molecular orbitals (MO) in the ground and excited states. These spectra have been compared with experiments, and the model structure shows the best similarities and good conformity. Supported by these results, a desirable charge/energy transfer occurred between the PVK and F8T2 blocks that could vary the optoelectronic properties and applications of this new copolymer with an electron donor/acceptor architecture. By coupling PVK to F8T2, a reduction of the electronic and optical gap was detected. The optical transitions of the PVK-F8T2 are now clear based on the DFT calculations. The complementarily experimental and theoretical calculations facilitate the study of the obtained copolymer and provide a better understanding of the properties of the PVK-F8T2 and their appropriate optoelectronic properties and application. In addition to the experimental and theoretical investigation of PVK-F8T2 with the aim of testing their effectiveness in the field of organic electronics, it can be said that the PVK-F8T2 has qualified properties that allow it to be exploited as an active layer in optoelectronic devices. As far as this study is concerned, further optimization of the multiple properties (electrical study, photoconductivity), eventual applications (OLED device) and nanowires (elaboration and study) are under study. In fact, the interaction and the charge transfer between the PVK-F8T2 and nanocarbon (carbon nanotube (CNT), C_60_, [6,6]-Phenyl C_61_ butyric acid methyl ester (PCBM), etc.) and their application is under study as well.

## Figures and Tables

**Table 1:** Assignments of the electronic transitions of the PVK-F8T2.

TD-DFT/6-31G (d,p)		λ _max_ (nm) (eV)	Force Oscillateur (f)	Assignement H,L (H = HOMO, L = LUMO)
Absorption	T1	222 Exp (221)	0.2260	H-2→L+8(+18%) H-1→L+8(7%) H-5→L+8(+7%) H-2→L+6(6%)
		5.58 Exp (5.61)		
	T2	252 Exp (254)	0.8169	H-3→L+4(+15%) H-4→L+9(+11%) H-1→L+2(+8%) H-1→L+6(+5%) H-7→L+0(+12%) H-0→L+6(7%)
		4.92 Exp (4.88)		
		306 Exp (312)	0.7990	H-3→L+2(+50%) H-1→L+4(7%)
		4.05 Exp (3.97)		
		358 Exp (361)	0.5777	H-0→L+1(+41%) H-2→L+0(14%) H-1→L+0(+7%) H-2→L+1(7%) H-1→L+1(+7%)
		3.46 Exp (3.43)		
	T3	507 Exp (504, 530, 563)	1.8032	H→L (86%)
		2.44 Exp (2.46; 2.33; 2.20)		

**Table 2:** Assignment of the PVK-F8T2 electronic transitions.

TD/DFT/6-31 G (d,p)		λ _max_ (nm)	Oscillator Force (f)	Assignment H,L (H = HOMO, L = LUMO)	τ_R_ (ns)
Emission	Excited state 1	499 Exp (510)	2.20	L→H (+98%) 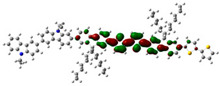 ↓ 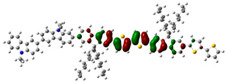	1.7
	Excited state 2	481.2 Exp (483)	1.14	L→H-1(+96%) 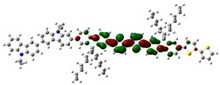 ↓ 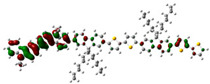	3.06
	Excited state 3	458 Exp (563)	0.86	L→H-2 (+52%) 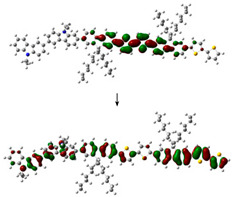 L+1→H-1(+6%) 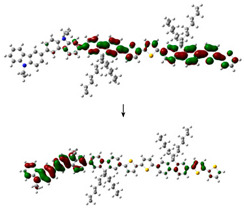 L→H-3 (+8%) 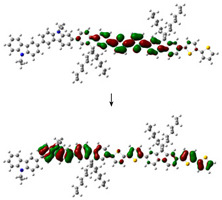	3.67

**Table 3:** Electron/holes injection parameters for PVK-F8T2 application in OLEDs [44,45].

	Φ_x_ (eV)	ΔE_h_ (eV)	ΔE_e_ (eV)	ΔE_e_ − ΔE_h_ (eV)
Au	5.30	0.06	3.42	3.36
Al	4.20	0.06	2.32	2.26
Mg	3.60	0.06	1.72	1.66
Ca	2.87	0.06	0.89	0.83

## Data Availability

Exclude this statement if the study did not report any data.
